# Blood Pressure Reducing Potential and Renoprotective Action of Cilnidipine Among Hypertensive Patients Suffering From Chronic Kidney Disease: A Meta-Analysis

**DOI:** 10.7759/cureus.37774

**Published:** 2023-04-18

**Authors:** Kusum Kumari, Ritesh Sinha, Mary S Toppo, Priyanki Mishra, Shadab Alam, Lakhan Majhee

**Affiliations:** 1 Pharmacology, Rajendra Institute of Medical Sciences, Ranchi, IND; 2 Pharmacology and Therapeutics, Rajendra Institute of Medical Sciences, Ranchi, IND; 3 Pharmacology, Mahatma Gandhi Medical College and Research Institute, Jamshedpur, IND

**Keywords:** blood pressure, proteinuria, chronic kidney disease, cilnidipine, hypertension

## Abstract

Hypertension is a risk factor for cardiovascular diseases which also causes progressive kidney damage leading to chronic kidney disease (CKD), so the rate of progression of CKD can be controlled by reducing blood pressure (BP). Many anti-hypertensive drugs are available. Cilnidipine is a new-generation calcium channel blocker (CCB). This meta-analysis is aimed to generate pooled evidence about the effectiveness of cilnidipine as an anti-hypertensive and to explore its reno-protective actions. Pubmed, Scopus, Cochrane Library, and Google Scholar were searched from January 2000 to December 2022 to include the studies. The pooled mean difference, along with 95% CI, was computed using Revman 5.4.1 software (Revman International, Inc., New York City, New York). The Cochrane risk-of-bias assessment tool was used for bias assessment. This meta-analysis was registered in PROSPERO with Reg. no. CRD42023395224. This meta-analysis included seven studies with 289 participants in the intervention group and 269 in the comparator group, and were selected from Japan, India, and Korea. Systolic blood pressure (SBP) was significantly reduced in cilnidipine treated group among hypertensives with CKD subjects weighted mean difference (WMD) was 4.33, and the 95% confidence interval (CI) was 1.26 to 7.31 as compared to the other group. Cilnidipine also shows a significant reduction in proteinuria with WMD 0.61 and 95% CI 0.42 to 0.80. Both groups were similar in adverse drug reactions (ADR). Cilnidipine is a more effective anti-hypertensive as compared to Amlodipine or other CCBs, mainly in reducing SBP. Besides this, cilnidipine also shows better reno-protective action because it also significantly reduces proteinuria in such patients.

## Introduction and background

Hypertension (HTN) is a worldwide problem and is also increasing day by day. It is a risk factor for not only cardiovascular diseases but also chronic kidney diseases (CKD). Initially, CKD classification was based only on glomerular filtration rate (GFR), but now proteinuria or the amount of albuminuria has also been incorporated in its definition. Kidney size of less than 10-15 cm in renal ultrasonography (USG) is suggestive of chronicity. Hypertension and CKD go hand in hand in the long run in life. Both are interrelated to each other. One of the major risk factors for developing CKD is HTN. So control of HTN is very important to slow the progression of CKD. Controlling blood pressure (BP) not only reduces CKD progression but also reduces cardiovascular risk [1]. Even CKD can be a cause of HTN. Currently, our treatment target for HTN control in CKD patients is systolic BP <130 mm Hg. Evidence shows that intensive BP control does not slow the rate of progress of CKD, but it reduces mortality in CKD patients [2-3]. 

The rate of progression of CKD can be controlled by reducing BP, so along with antihypertensive drug treatment, regular BP monitoring is also essential. Evidence shows that there are more chances of nocturnal HTN in hypertensive patients with CKD as compared to HTN without CKD. So nocturnal BP measurement is also important, which is only possible through ambulatory BP monitoring [4].

Different pharmacological treatments for HTN are available. Among various groups of antihypertensive drugs, diuretics, angiotensin-converting enzyme (ACE)-inhibitors, angiotensin receptor antagonists, calcium channel blockers (CCBs), vaso dilators, centrally acting alpha agonists, and β-blockers are commonly used in our community [5]. The renin-angiotensin-aldosterone system (RAAS) plays a very important role in the regulation of BP. So, the drugs acting on RAAS are the most widely used antihypertensive drugs. The final blockade of angiotensin 2 causes dilatation of efferent arterioles, which is responsible for its reno-protective action [5]. These drugs cause hyperkalemia and unproductive cough as side effects. Loop diuretics are preferred over thiazides when serum creatinine is more than 2.5 mg /dl [2], but they are also not safe for long-term use as fluid and electrolyte disturbances are the common side effects of diuretics. β blockers are alternative drugs, but there is a lack of evidence that β-blockers are more useful in CKD patients.

CCBs are another class of drugs for HTN. They block voltage-gated calcium channels (CC). Chemically, amlodipine is a dihydropyridine (DHP), which blocks L-type voltage-gated CC. Blockade of these channels causes vasodilatation. They show more affinity for plasma proteins, so they are mainly metabolized in the liver. Their dose modification is not required in kidney dysfunction patients.

Another type of CC is N-type channels which are present in the brain and nerves. Cilnidipine blocks both N as well as L-type calcium channels. By blocking these channels, cilnidipine suppresses the hyperactivity of the sympathetic nervous system (SNS) and RAAS. Thereby it might show some reno-protective actions [6]. Nowadays, cilnidipine and amlodipine are both used as antihypertensive drugs, but whether cilnidipine is comparable or is a more efficacious drug than amlodipine or any other L-type CCB has not been established yet. There are few studies that show the efficacy of cilnidipine among hypertensive patients with CKD, but their results are very inconsistent. So to generate better evidence, meta-analyses of these studies are necessary so that our physicians can choose a better antihypertensive drug for CKD patients. This meta-analysis will generate pooled evidence on whether cilnidipine is effective in those hypertensive patients and also for its renal-protective effect.

## Review

Methodology

Study Selection Process

The guidelines of Preferred Reporting Items for Systematic Reviews and Meta-Analyses (PRISMA) have been followed for preparing this meta-analysis. Two independent reviewers searched Pubmed, Scopus, Cochrane Library, and Google Scholar to search for the required studies from Jan. 2000 to Dec. 2022. These studies were searched using the keywords hypertension or cilnidipine OR reno-protective action.

Research Question

The study included a population of hypertensive adult patients suffering from CKD for more than three months. The intervention group received treatment with cilnidipine, while the comparative group was treated with any other CCB except cilnidipine. The outcomes were mean systolic and diastolic BP reduction, decrease in serum creatinine or urinary protein/creatinine ratio, and estimated GFR along with any adverse outcome. Eligibility criteria have been shown in Table 1.

**Table 1:** Eligibility criteria CKD - chronic kidney disease, SBP - systolic blood pressure, DBP - diastolic blood pressure

Criteria	Criterion
Inclusion criteria	All randomized controlled trials (RCT) and retrospective studies including adult hypertensive subjects suffering from CKD with an age of more than 18 years.
	Experimental group treated with at least cilnidipine and a comparator group without cilnidipine.
	Study duration of more than 12 weeks
	Studies with sufficient data on the reduction of SBP and DBP along with renal function parameters or any adverse drug reaction (ADR)
Exclusion criteria	Clinical trials in which a comparable group was not mentioned.
	Experimental group not receiving cilnidipine.
	Study in which no renal parameters were given.
	Insufficient data reported to analyze pooled effect.

Data Extraction

Two independent reviewers extracted the data from the included studies. Data was cross-checked for consistency, and any difference in data was corrected by rechecking and discussion. Data was collected in the form of the year of publication, author name, sample size, study population, any comorbidity, mean age of study participants, drugs used in the intervention and comparator group, and the outcome parameters. Any adverse drug reaction (ADR) data was also reported.

Statistical Analysis

Continuous data was computed in the form of weighted mean difference (WMD) with 95% confidence interval (CI). In case of significant heterogeneity (chi²>50%), the random effect model was used, while a fixed effect model was used for homogeneous data. All the continuous variable was presented along with standard deviation (SD). In case of heterogeneity, STATA software (StataCorp LLC, College Station, Texas) was used to explore the cause of heterogeneity. Dichotomous data was also collected with 95% CI. All the data was entered in Revman 5.4.1 (Revman International, Inc., New York City, New York), and pooled analysis was done.

Bias Assessment Tool

Bias assessment of all the included randomized controlled trials (RCTs) was done using the Cochrane risk-of-bias assessment tool. Six domains of this tool were assessed by two different reviewers independently. For assessing selection bias, random sequence generation and allocation concealment were checked. Performance bias was assessed by checking the blinding procedure used for participants and other personnel. Detection bias was assessed by observing the blinding status of the outcome assessor. The above domains were categorized into low-risk and high-risk. Reporting bias was assessed based on the completeness of outcome data. If all the given parameters were reported as mentioned in the methodology section, then it was low-risk; otherwise, it was considered as high-risk bias. Lastly, attrition bias was assessed based on the number of losses to follow-up participants. If less than 10% loss to follow-up, it was low-risk, and if more than 10%, then it was high-risk. Bias assessment was done using Revman software 5.4.1.

This study has been registered in PROSPERO with registration number CRD42023395224.

Results

Study Selection Process

**Figure 1:**
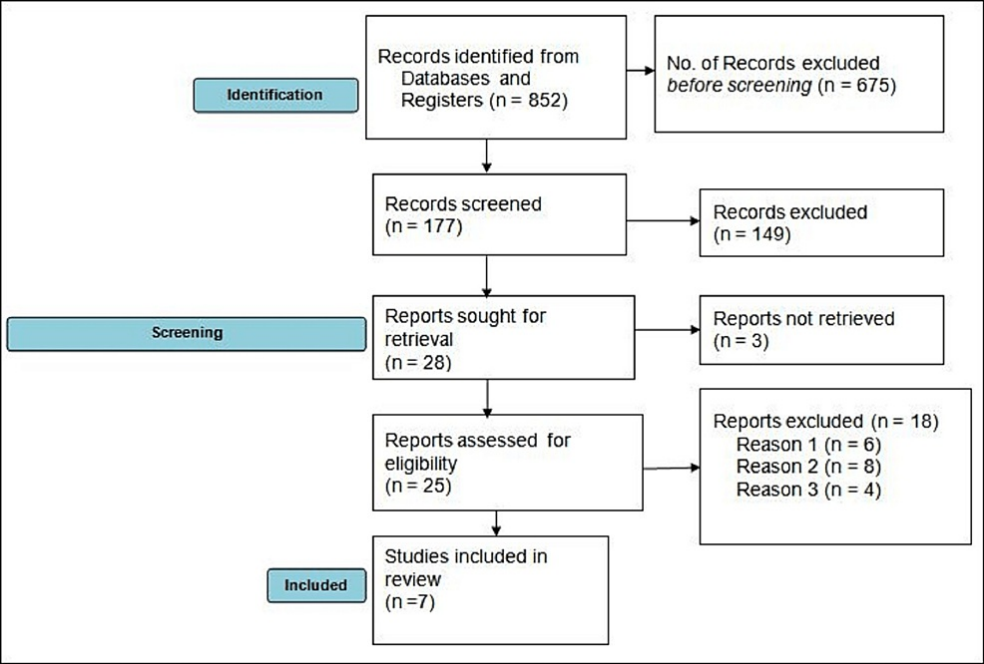
Study selection process using PRISMA guideline PRISMA - Preferred Reporting Items for Systematic Reviews and Meta-Analyses

By using different databases and registers, a total of 852 studies were identified. Before screening, 675 studies were excluded due to irrelevant studies. One hundred forty-nine were again excluded after screening as they did not match our objectives. A further three studies could not be retrieved so among 25 eligible studies, 18 were again excluded due to not meeting inclusion criteria, no proper data outcome, and the absence of any comparable group. Finally, seven eligible studies [7-13] were used for pooled analysis. The study selection process is shown in Figure 1.

Characteristics of Included Studies

Details of the selected studies are shown in Table 2. Among seven studies, five were conducted in Japan, out of which one study was multicentric and four were single-centered. One study was conducted in India and one in Korea. Study duration ranges from 24 weeks to 12 months. In all the studies, experimental arms were treated with cilnidipine, while the comparator arm was treated with CCB other than cilnidipine. Six studies were open-label RCT, while one was a retrospective study.

**Table 2:** Included study characteristics RCT - randomized controlled trial, m - month, CRF - chronic renal failure, CKD - chronic kidney disease, HTN - hypertension, CRD - chronic renal disease, L.CCB - L-type calcium channel blocker, SBP - systolic blood pressure, DBP - diastolic blood pressure, S creatinine - serum creatinine, U protein - urinary protein, GFR - glomerular filtration rate, ADR - adverse drug reaction, EGFR - estimated glomerular filtration rate, UPCR - urinary protein creatinine ratio, U. albuminuria - urinary albuminuria

Year	Author	Type of study	Site	Duration	Sample size	Study population	Co-morbidity	Mean age	Drug in intervention	Drug in comparator	Outcome parameters
2004	Kojima et al. [13]	Open, RCT	Japan	12 m	28	Hypertension	CKD	62 yrs	Cilnidipine	Amlodipine	SBP, DBP, S creatinine, U protein
2007	Fujita et al. [7]	Open, RCT multicentric	Japan	12 m	339	Hypertension	CKD	59.6 yrs	Cilnidipine	Amlodipine	SBP, DBP, GFR, ADR, U protein
2009	Satomora et al. [8]	Open, RCT	Japan	12 m	33	CRF		63 yrs	Cilnidipine	Amlodipine	SBP, DBP, GFR, U protein
2012	Hatta et al. [12]	Open, RCT	Japan	12 m	50	CKD		56.5 yrs	Cilnidipine	L.CCB	SBP, DBP, S creatinine, GFR, U Protein
2013	Kanaoka et al. [10]	Open, RCT	Japan	24 weeks	45	HTN	CKD	69.9 yrs	Cilnidipine	CCB	SBP, DBP, S creatinine, GFR, UPCR
2013	Mallashappa et al. [9]	Open, RCT	India	6 m	60	HTN	CKD	44.45 yrs	Cilnidipine	Others	SBP, DBP, S creatinine, GFR, U. albuminuria
2020	Oh et al. [11]	Retrospective	Korea	12 m	53	CKD		59.4 yrs	Cilnidipine	Efondipine	SBP, DBP, S creatinine, U protein, GFR

Primary Outcome

**Figure 2:**
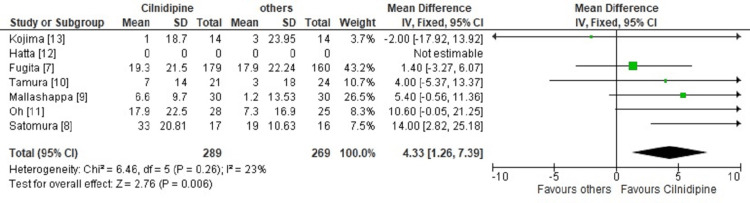
Comparison of mean systolic blood pressure reduction

The primary outcome of this meta-analysis was a reduction in the mean SBP and DBP among study participants. We got reductions in both SBP and DBP in the cilnidipine-treated arm as compared to the comparator arm, but SBP reduction was significant with a weighted mean difference (WMD) of 4.33 and 95% confidence interval of 1.26 to 7.39 as shown in Figure 2. There was some heterogeneity, but it was not significant (Chi^2^=6.46, I2=23%, p=0.26).

DBP reduction was not significant by cilnidipine with WMD 1.74 and 95% CI -0.15 to 3.62. No heterogeneity was seen.

Secondary Outcome

Changes in different renal parameters were our secondary outcome. Insignificant change in serum creatinine was seen in cilnidipine treated group as compared to the comparator group, with WMD -0.02 and 95% CI -0.11 to 0.08, which indicates that serum creatinine level increases by cilnidipine. Similarly, insignificant change was found in the GFR level also, where WMD was 0.75 with 95% CI -1.66 to 3.16, as shown in Table 3.

**Table 3:** Comparison of different outcome parameters DBP - diastolic blood pressure, GFR - glomerular filtration rate, WMD - weighted mean difference, CI - confidence interval

Outcome parameter	No. of studies	WMD; 95% CI	Test of heterogeneity	Overall effect
DBP	6	1.74; -0.15, 3.62	Chi^2^=4.92; I^2^=0%	Z=1.80; p=0.07
Serum creatinine	6	-0.02; -0.11, 0.08	Chi^2^=4.92; I^2^=0%	Z=0.31; p=0.76
GFR	3	0.75; -1.66, 3.16	Chi^2^=0.32; I^2^=0%	Z=0.61; p=0.54

**Figure 3:**
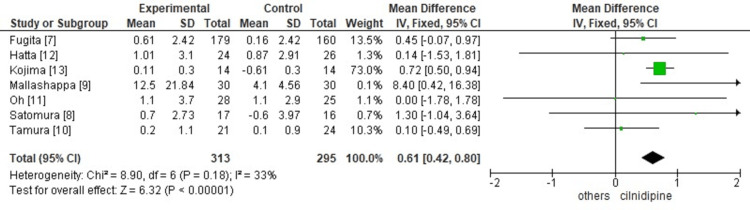
Comparison of decrease in proteinuria

We found a significant decrease in proteinuria with WMD 0.61 and 95% CI 0.42 to 0.80. Thirty-three percent heterogeneity was seen with Chi^2^=8.90 and p<0.00001. Seven studies were included, as shown in Figure 3.

**Figure 4:**
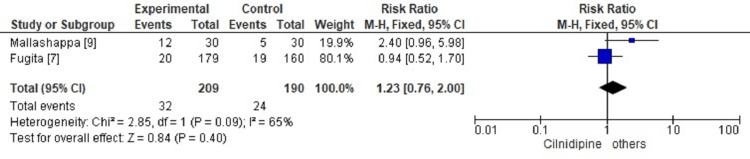
Comparison of adverse drug reactions

Two studies reported ADR (Figure 4), which depicts a risk ratio of 1.23 with a 95% CI of 0.76 to 2.00. A 2.68% increase in ADR was seen in the cilnidipine-treated group as compared to the comparator group, but it was insignificant, as indicated by a p-value of 0.40, and 65% heterogeneity was seen.

Risk-of-Bias Assessment

**Figure 5:**
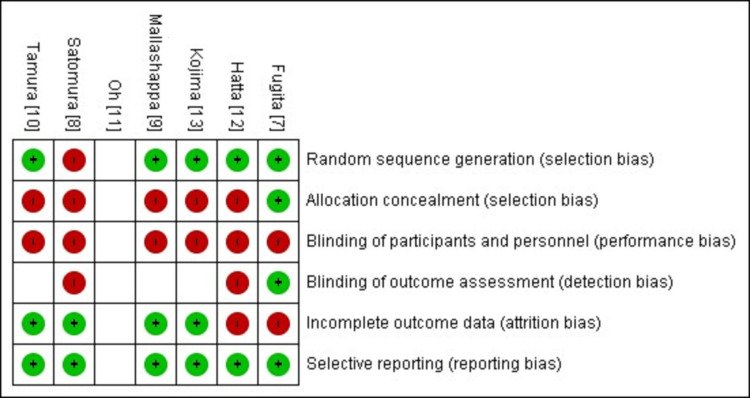
Bias assessment table

Five studies showed low risk in random sequence generation, while one study showed high risk. In only one study, allocation concealment was followed. All the included studies showed high risk of performance bias because all were open-label studies. Only one study showed low risk in detection bias; three showed unclear risk, while two showed high risk (Figure 5). In two studies, the loss of follow-up data was more than 10%, so these studies were categorized as having a high risk of attrition bias.

**Figure 6:**
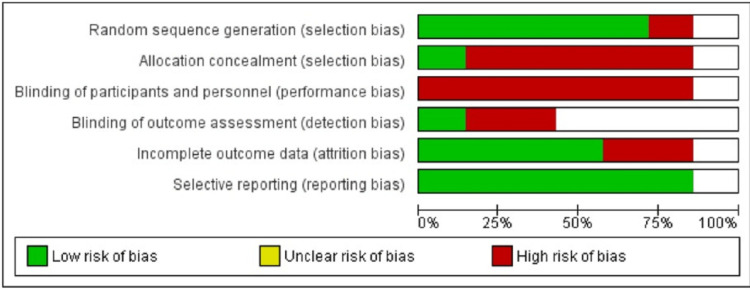
Risk-of-bias assessment graph

Surprisingly, all the studies showed a low risk of reporting bias. The risk-of-bias assessment graph is shown in (Figure 6), which shows a 100% high risk in performance bias.

Discussion

In this meta-analysis, seven studies from different countries ( Japan, India, and Korea) were included, with 289 participants in the cilnidipine-treated group and 269 in the comparator group. The study participants were those hypertensive patients who were also suffering from CKD for more than three months. Our pooled analysis showed that cilnidipine decreased mean SBP significantly with an insignificant lowering of mean DBP value. Our finding is different from the previous meta-analysis conducted among hypertensive Chinese patients, which showed that there was no difference in cilnidipine and amlodipine among mild to moderate essential hypertensive patients [14]. This difference in findings must be due to a difference in the genetic makeup of the included participants. Another reason may be due to the different inclusion criteria used. We included hypertensive patients with CKD, while in a previous study, all participants were mild to moderate essential hypertensives without CKD.

Cilnidipine and amlodipine are both CCBs belonging to the DHP group. Both block L-type voltage-gated CC, but cilnidipine additionally blocks N-type CC also [15]. Due to the presence of N-type channels along the nerves, cilnidipine attenuates norepinephrine release from sympathetic nerve endings. So cilnidipine causes more reduction in SBP and DBP as compared to amlodipine.

Our pooled analysis also shows that cilnidipine does not significantly change serum creatinine levels as well as GFR. But this study provides evidence that cilnidipine has renal protective actions as it significantly decreases proteinuria in CKD patients, which supports the previous meta-analysis in which renal function was assessed among hypertensive patients receiving cilnidipine and L-type CCBs and cilnidipine showed better improvement in urinary protein excretion with similar effect on serum creatinine[16]. It can be explained by N-channel blocking the action of cilnidipine. So, it inhibits glomerular HTN by reducing SNS discharge. Amlodipine selectively dilates only afferent glomerular arterioles causing intraglomerular HTN. But cilnidipine, on the other hand, can dilate both afferent and efferent arterioles, thereby, does not produce glomerular HTN, and thus the glomeruli are protected[17]. Besides this, cilnidipine also suppresses RAAS and so decreases oxidative stress. Anti-oxidative property of cilnidipine also enhances its reno-protective action due to the protection of podocytes[18]. Excess reactive oxygen radicles may initiate the development of different renal diseases. As cilnidipine can reduce oxidative stress thereby, it shows a greater reno-protective effect than amlodipine [18]. Cilnidipine is also more lipophilic, which increases its bioavailability.

Renoprotective action of cilnidipine has been shown in hypertensive rats also, in which HTN was induced by oral administration of one of the L-arginine analogs in distilled water for four weeks[19]. Interestingly, cilnidipine also lowers the triglyceride level, which is again beneficial for hypertensive subjects because dyslipidemia causing endothelial dysfunction is an independent risk factor for HTN. Cilnidipine has alpha receptor blocking action also, which causes peripheral vasodilation, which enhances glucose and lipid uptake by muscle cells [20].

Blocking of N-type CC provides additional organ protective activity to cilnidipine, like cardio-protection and reno-protection [21]. One study among subjects with proteinuria also shows that cilnidipine is a better anti-hypertensive and reno-protective drug than amlodipine [22]. In another study conducted among diabetic hypertensives, cilnidipine shows similar BP-reducing potential as olmesartan, although both drugs act by different mechanisms; however, cilnidipine reduces micro-albuminuria more effectively than olmesartan [23]. Regarding ADR, the main ADR of amlodipine is ankle edema, whereas cilnidipine has the potential to reduce this pedal edema in subjects treated with amlodipine. So cilnidipine can be used as an alternative drug to amlodipine, especially in patients with pedal edema[24].

Novelty

This is the first time the anti-hypertensive action of cilnidipine among hypertensive patients with CKD in Jharkhand has been assessed. In addition to this, its renal protective action was also assessed for the first time among CKD patients, and a meta-analysis was done, which will provide better evidence for choosing the better drug for treating physicians, especially for CKD patients.

## Conclusions

Our pooled analysis shows that cilnidipine is more effective than amlodipine in reducing SBP among patients with CKD. Cilnidipine is a more lipophilic drug than amlodipine, so its bioavailability increases. Cilnidipine has an inhibitory action on SNS, which increases its BP-reducing potential. Its alpha-blocking action also helps in BP reduction; thereby, it can be a better choice than amlodipine as an antihypertensive drug.

Further, cilnidipine also significantly decreases proteinuria in CKD patients, so it shows its renal protective actions. It also inhibits RAAS, which adds to its renal protective action. So cilnidipine can be preferred over amlodipine or other L-type CCBs, especially in CKD patients, because it can better control BP as well as proteinuria. It has the potential to prevent the progression of CKD, which is very important. It is also advantageous because it also lowers the triglyceride level, which is again helpful in reducing mortality among hypertensive subjects.
